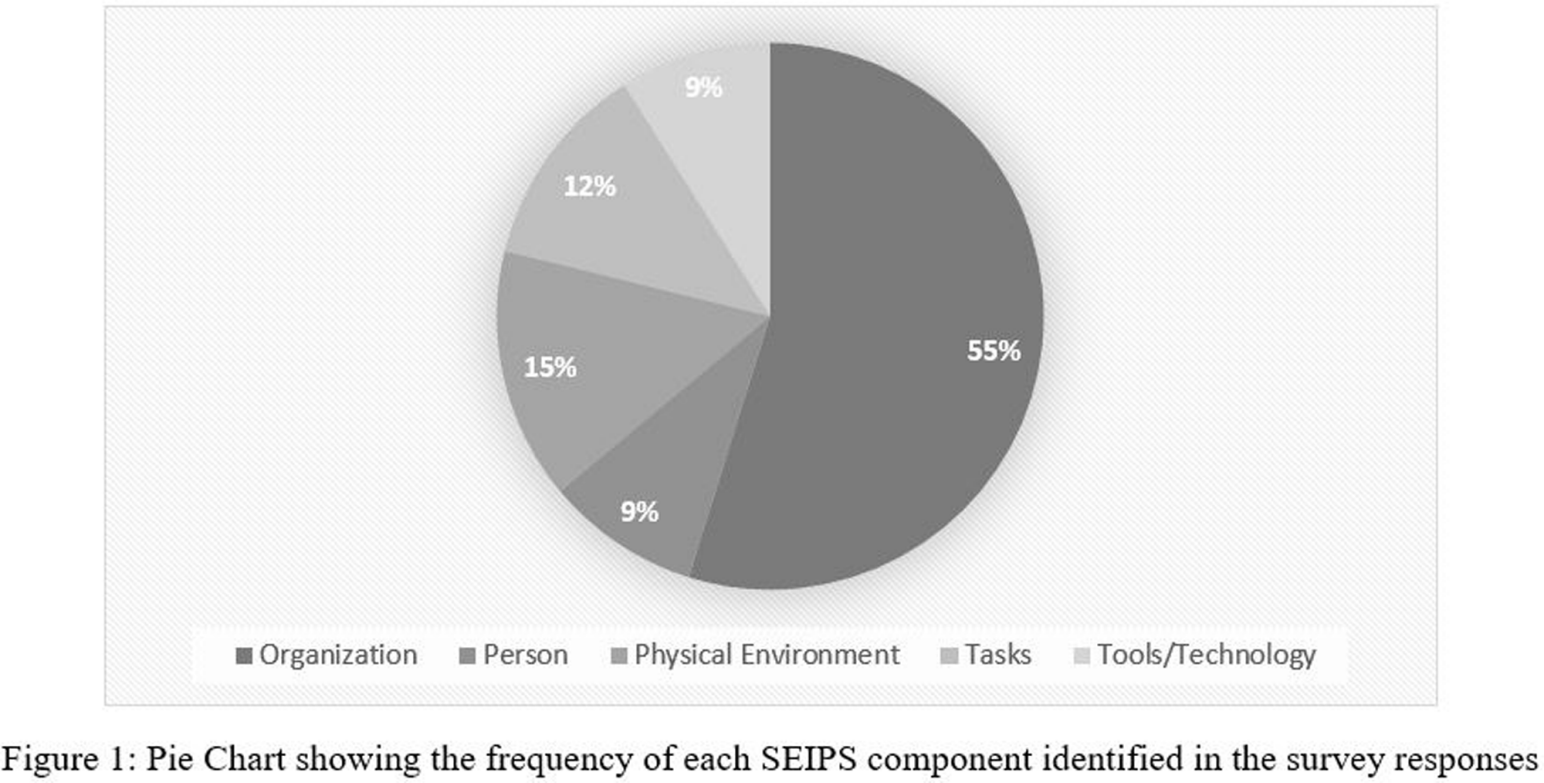# Presenteeism Among Healthcare Professionals (HCP) During the COVID-19 Pandemic: Survey of Perceived Barriers

**DOI:** 10.1017/ash.2024.251

**Published:** 2024-09-16

**Authors:** Katherine Dolan, Rachel Meyer, Laura Anderson, Dan Shirley, Michael Kessler, Linda Stevens, Nasia Safdar

**Affiliations:** University of Wisconsin School of Medicine and Public Health; University of Wisconsin Hospital and Clinics; UW Health; UW Madison School of Medicine and Public Health; University of Wisconsin, Madison

## Abstract

**Background:** Presenteeism when ill in healthcare personnel (HCP) can contribute to the spread of respiratory illness among HCP and patients. However, during the COVID-19 pandemic and now, there are substantial challenges preventing HCP from staying home when ill. We examined these challenges using the Systems Engineering Initiative for Patient Safety (SEIPS) framework. **Method:** As part of a larger anonymous electronic survey between 3/11/2022 and 4/12/2022 at an academic tertiary referral center, in inpatient and ambulatory settings where respondents were asked to describe factors impacting presenteeism when ill, we analyzed free-text responses using the SEIPS categories of tasks, tools/technology, person, organization, and physical environment. **Result:** 522 comments were received in response to the open-ended survey question asking individuals to describe any factors that would assist them in remaining home and/or help them get tested for COVID-19 when they have symptoms of a respiratory illness; 21 were excluded due to absent or incomplete response. Of the remaining responses (N = 501, Figure 1), 82% were associated with a single SEIPS component such as organization (N = 409), while other responses discussed factors that involved two SEIPS components, in no particular order (N = 92). A majority of the responses (N = 324, 55%) reported organizational barriers, frequently citing a strict sick call-in policy as well as a lack of protected time-off for COVID-19 testing or related absences. The next two most commonly identified components were physical environment (N= 88, 15%) and tasks (N = 72, 12%), mentioning barriers such as far distances to testing centers and prolonged waiting periods for testing **Results:** The person and tools/technology components were less commonly identified, with a frequency of 9% each. **Conclusion:** A number of systems level factors were identified that may impact the ability of HCP to stay home when ill. Interventions to help overcome HCP perceived barriers to staying home when experiencing respiratory symptoms should focus on the policies and practices within an organization. Communication from leadership should support staying home with respiratory symptoms by creating plans for coverage and back up consistently across all employee types in direct care.

**Figure:**